# The World's Scientific Authority and Health-Related Challenges 

**Published:** 2017-04-17

**Authors:** Jalal Poorolajal

**Affiliations:** ^1^ Research Center for Health Sciences, Department of Epidemiology, School of Public Health, Hamadan University of Medical Sciences, Hamadan, Iran.

Iran has made a substantial progress in science and technology during the last two decades, despite international sanctions. Iran's scientific community remained productive, even while economic sanctions make it difficult for Iranian universities to prepare their requisite equipment^1^. Furthermore, Iran's university population has swelled from 100,000 in 1979 to more than 4.5 million in 2012^2, 3^.

**Figure 1:**
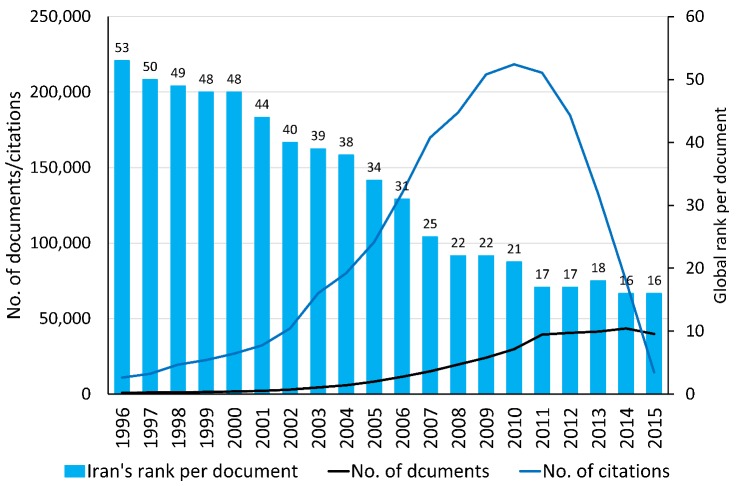
Number of Iran's scietitifc productions and citations and global rank per scietific production during the last two decades

**Figure 2:**
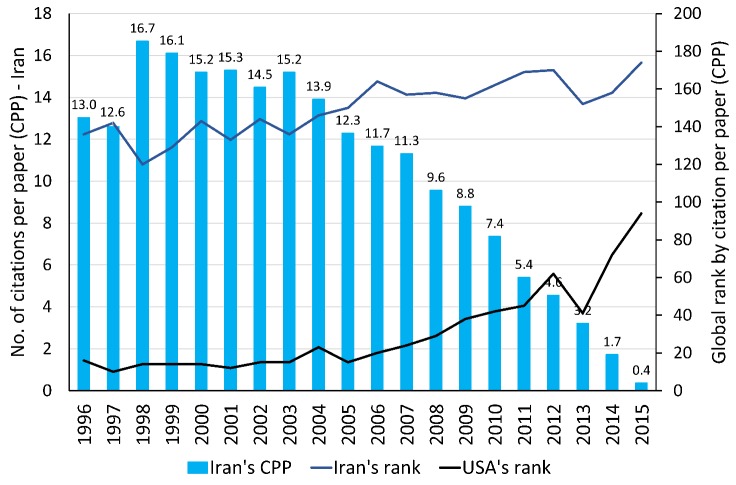
Number of Iran's citation per paper (CPP) and comparison of Iran's and the United States' global rank by CPP during the last two decades

Based on the Scopus database^4^, the number of citable documents produced by Iranian scientists rose about 48-fold during the last two decades, from 832 citable documents in 1996 to 39,727 in 2015. According to this database, Iran produced only 0.07% of the scientific products of the world in 1996, but 1.32% in 2015. Iran could improve its scientific level among the world's top scientific countries during the last two decades and could pass many developed countries of the world and reach the 16th global rank and the first regional rank in 2015 (Figure 1). This figure indicates an increasing trend in the number of citations to Iranian scientific production from 1996 to 2010 and a decreasing trend thereafter. In the first view, one may imagine that the quality of the Iranian scientific productions has fallen in the recent years and the number of citations to Iranian publications has not been growing along with the number of scientific products. However, we should keep in mind that old papers had more opportunity to be seen, read, and cited than the new ones. That means citation to scientific productions is a time-dependent process. As shown in Figure 2, during the last two decades, the global rank of citations per paper (CPP) for Iran changed in the same way as it did for the United States.

**Figure 3:**
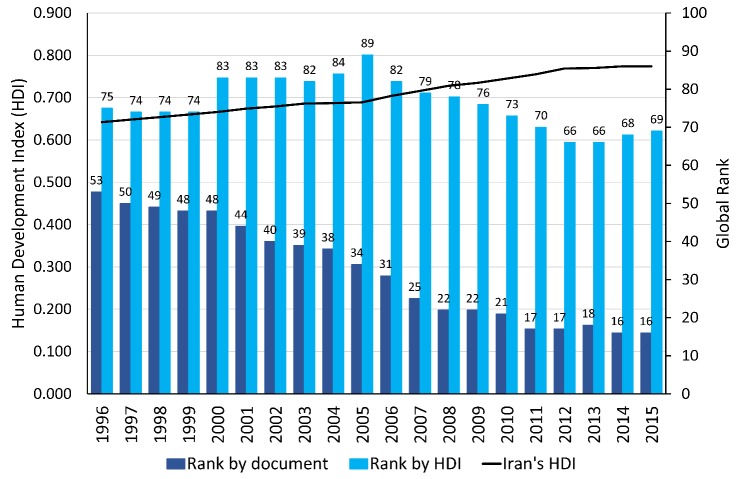
Iran's human developmet index (HDI) and global rank by HDI and scientific productions during the last two decades

Based on the Iranian 'Comprehensive Scientific Map' (CSP), Iran must achieve the first place of science and technology in the Muslim world and obtain an outstanding position of science and technology in the world by 2025^5^. In addition, Iran should achieve the authority of science and technology in the world by 2055. That means experts and scientists of the world need to refer to Iranian publications. For this purpose, we have to be a pioneer in many aspects of science and technology as well as the human resources. As shown in Figure 3, Iran's human development index (HDI) value has been growing slowly during the last two decades^6^. Based on this figure, Iran's scientific productions have been improving constantly over time, however, HDI has not been improving along with growing in the number of scientific productions during the last two decades. As shown in Table 1, Iran could achieve an outstanding position related to scientific productions among developed countries, but Iran is still far from its proper position in health-related indicators such as HDI, gender development index (GDI), total disability adjusted life year (DALY), life expectancy at birth, maternal mortality rate, and under-5 mortality rate. Healthy and productive human resources is the best and most valuable asset of every country. Therefore, progress will not be achieved unless special attention is paid to this brilliant asset.

**Table 1:** Iran's rank related to health indicators

**Indicators**	**Year**	**Esimate**	**Rank**
Sientific productions^4^	2015	39,727	16
Citation per document^4^	1996-2015	5.86	201
Human development index^6^	2015	0.774	69
Gender development index^7^	2015	0.862	69
Total DALY rate (Í100,000)^ a8^	2014	30911	81
Life expectancy at birth^9^	2015	75.5	60
Maternal mortality rate (Í100,000)^ a10^	2015	25	61
Under-5 mortality rate (Í1000)^ a11^	2015	16	86

^a^ Ranked from the lowest rate to the highest

In order to achieve an outstanding place of science and technology of the world and the world's scientific authority, Iran requires to improve not only the health status of its human resources, but also the infrastructure of the science and technology on the basis on what mentioned in the CSP^5^ as follows. About 7% of the growth domestic product (GDP) must be dedicated to education and 7% to research ($37,107,000,000 and $16,492,000,000, respectively based on GDP in 2016). The number of full-time faculty members must increase to 2000 per million populations (i.e., 160,000 faculty members based on 2016 population). Full-time researchers must include 10% of the government and 50% of the higher educational institutes. The number of papers per population must be 800 per one million populations. The number of CPP must be 15 per paper. About 4% of the annual GDP growth must result from science and technology, etc. Until requirements for growth and development are provided and essential infrastructures are improved, it is difficult to achieve the world's scientific authority.
